# Phytochemical Enhancement in Broccoli Florets after Harvest by Controlled Doses of Ozone †

**DOI:** 10.3390/foods11152195

**Published:** 2022-07-23

**Authors:** Arturo Duarte-Sierra, Charles F. Forney, Minty Thomas, Paul Angers, Joseph Arul

**Affiliations:** 1 Food Science Department, Laval University, Quebec, QC G1V 0A6, Canada; minty.thomas.1@ulaval.ca (M.T.); paul.angers@fsaa.ulaval.ca (P.A.); joseph.arul.1@ulaval.ca (J.A.); 2 Institute on Nutrition and Functional Foods (INAF), Laval University, Quebec, QC G1V 0A6, Canada; 3 Center for Research in Plant Innovation (CRIV), Laval University, Quebec, QC G1V 0A6, Canada; 4 Kentville Research and Development Centre, Agriculture and Agri-Food Canada, 32 Main Street, Kentville, NS B4N 1J5, Canada; charles.forney@canada.ca

**Keywords:** broccoli, O_3_, oxidative stress, glucosinolates, hydroxy-cinnamates

## Abstract

The objective of this work was to examine the effect of controlled doses of O_3_ (0, 5 µL L^−1^ of O_3_ for 60 min, and 5 µL L^−1^ of O_3_ for 720 min) on the quality and phytochemical content of broccoli florets during postharvest storage. The optimal dose was found at 5 µL L^−1^ of O_3_ for 60 min, from the color retention of broccoli florets exposed to the gas treatment. Overall, the antioxidant capacity of the florets was significantly affected by both doses of O_3_ compared to the non-exposed florets. The profile of glucosinolates was determined for up to 14 days in broccoli florets stored at 4 °C by LC-MS. The amount of total glucobrassicins and total hydroxy-cinnamates in florets significantly (*p* ≤ 0.05) improved by the application of 5 µL L^−1^ of O_3_ for 60 min compared to non-treated florets. The up-regulation of genes of the tryptophan-derived glucosinolate pathway was observed immediately after both treatments. The gene expression of CYP79A2 and CYP79B3 in broccoli was significantly higher in broccoli florets exposed to 5 µL L^−1^ of O_3_ for 720 min compared to non-exposed florets. Although enhancement of secondary metabolites can be achieved by the fumigation of broccoli florets with low doses of ozone, quality parameters, particularly weight loss, can be compromised.

## 1. Introduction

Ozone (O_3_) is an allotrope of oxygen (O_2_), and a strong oxidizing agent that produces free radicals and harmful effects to living organisms at high concentrations. In humans, ozone can cause disruption of the cell signaling in the respiratory tract, increase the heart rate, and cause vascular oxidative stress [1]. Ozone can also react with plant cell structures, including cell membrane lipids, proteins, nucleic acids, olefinic compounds of the cuticle, and phenolic compounds [2,3]. As O_3_ diffuses into the intercellular space, ascorbic acid may limit the amount of the gas penetrating through the cell wall, avoiding the contact with more vulnerable structures inside the plasmalemma [4]. Evidence suggests that plants protect themselves from O_3_ by accumulating ascorbic acid in the cell walls that ultimately limits the entrance of the gas to more vulnerable structures [4]. Along with ascorbic acid, other antioxidants present in the apoplast are phenols and sulfhydryl amino acids [5]. Furthermore, O_3_ influences the antioxidant enzymatic system of the cell including superoxide dismutase, catalase, glutathione peroxidase as well as ascorbate peroxidase [6].

The biochemical responses induced by O_3_ include the induction of polyamine and ethylene biosynthesis [7]. The induction of stilbene biosynthesis has also been observed in conifer species after exposure to O_3_. An increase in the activities of phenylalanine ammonia-lyase and chalcone synthase was observed prior to the biosynthesis of the stilbenoids, pinosylvin and pinosylvin 3-methyl ether [8]. The exposure of plants to O_3_ has been found to cause similar responses to that of a pathogen attack. The induction of β-1,3-gluconate and chitinase in leaf cells, and the production of necrotic spots by ozone, have been observed [9].

Contrary to plants, where atmospheric concentrations cannot be controlled, in crops, it is intentionally applied at specific amounts to serve as disinfectant agent, since it is very effective against microorganisms, and it does not leave any residues in their surface [10]. The gasification of O_3_ on produce has been gaining attention as a disinfectant agent for produce since 2001 when the U.S. Food and Drug Administration (FDA) recognized its use as antimicrobial agent for the treatment, storage, and processing on foods in gas and aqueous phases. Since then, the effectiveness of O_3_ fumigation, as well as its incorporation with water, have been tested on different products and phytopathogens [11,12,13].

Although O_3_ is mainly used for disinfection purposes, its capacity to destroy ethylene makes this gas ideal for the storage of fresh produce when appropriate doses are used. In addition to these applications, the research in postharvest during recent years has moved into the effects of O_3_ inducing the antioxidant and phytochemical composition in produce [14,15]. The information regarding the induction of the beneficial effects of O_3_ exposure in postharvest is limited, but some examples are available. Ozone can activate defense mechanisms through gene expression and the accumulation of small molecules with a protective action: α-tocopherol and sinigrin in cabbage [16]. The secondary metabolites with protective roles vary according to the species, and one of the most abundant classes is that of phenolic compounds; thus, for example, it has also been observed that ozone at 0.1 µL L^−1^ increased the total flavan-3-ol content, maintained the levels of hydroxycinnamates, and increased the total phenolics in table grapes [17]. Moreover, at preharvest, an induction of glucobrassicins (indole-type glucosinolates) using ozonated water at 0.2 mg L^−1^ has already been observed [18]. However, it is necessary to be particularly careful when selecting the dosages, due to the powerful oxidative properties of this molecule, which can react with virtually all components of the cells, causing significant damage.

Ozone at low doses may elicit adaptive or beneficial processes in biological systems (a phenomenon known as hormesis) [19,20]. UV-C hormesis has been shown in many postharvest crops, and is known to induce disease resistance and delayed senescence responses in fresh fruits and vegetables [21,22]. However, it is not known whether such a hormetic phenomenon exists with ozone exposure in postharvest systems. Thus, the objective of this work was to determine hormetic doses of O_3_ in terms of color retention. In addition, the effect of hormetic and high doses (5 µL L^−1^ of O_3_ for 60 min, and 5 µL L^−1^ of O_3_ for 720 min) on the quality and the evolution of secondary metabolites, glucosinolates, and hydroxycinnamic acids in broccoli florets during storage was determined. Furthermore, the gene expression of some of the key enzymes in glucosinolate and phenylpropanoid pathways in broccoli exposed to theses stresses was also monitored.

## 2. Materials and Methods

### 2.1. Broccoli

Fresh mature heads (0.6 kg, 0.1–0.15 m) of broccoli (*Brassica oleracea* L. var. italica ‘Diplomat’) were acquired from packaging houses on the Island of Orleans, Quebec, Canada. Floret selection was earlier discussed by Duarte-Sierra et al. [23] and Duarte-Sierra et al. [24]. In brief, florets were separated from heads using a surgical blade, and stored in dark conditions at 4 °C/90–95% RH for 12 h to minimize the wound stress response before applying O_3_ treatments.

### 2.2. Determination of the Hormetic Dose

Ozone treatment was carried out in an airtight humidified (70–80%) plexiglass chamber (1 m ×1 m ×1 m) at 10 °C. Ozone gas was generated by corona discharge (SF300, Burlington, ON, USA), and the concentration was measured by an O_3_ detector (IN-2000, InUSA Inc., Needham, MA, USA) and controlled with a computer 21× Micrologger (Campbell Scientific, Logan, UT, USA). Treatments were performed using 5 µL L^−1^ of O_3_ at different times (0–720 min). The exposure times using 5 µL L^−1^ of O_3_ were 0, 7.5, 15, 30, 60, 120, 240, 480, and 720 min. The hormetic dose selection was based on the minimal total color change (ΔE) of florets (*n* = 9) at the end of the storage of 21 days.

The storage conditions for further treatments consisted of 30 day at 4 °C and 90% RH. Samples were drawn on 0, 1, 2, 4, 7, 14, and 21 days for further analysis.

### 2.3. Color, Respiration Rate, and Weight Loss Measurements of Florets during Storage

A detailed description of this methodology has been given previously [23,24]. The color of broccoli florets was measured using a colorimeter set with a D 65 illuminant (Minolta CR200, Osaka, Japan) using CIELAB color space (i.e., L*, a*, and b*). The color was determined on 9 florets samples from each treatment daily for 21 days of storage. The total color change (ΔE) was calculated from L*, a*, and b* values using the following equation:

$$\sqrt{{\left(L_{0}^{*}-L_{t}^{*}\right)}^{2}+{\left(a_{0}^{*}-a_{t}^{*}\right)}^{2}+{\left(b_{0}^{*}-b_{t}^{*}\right)}^{2}}$$

The respiration rate (nmol kg^−1^ s^−1^) of broccoli florets was analyzed by measuring concentrations of CO_2_ and O_2_ using a headspace analyzer on trigger mode (CheckMate 9900, Cambridge, ON, Canada). Along with the percentage of weight loss, the respiration rate of broccoli florets was assessed in triplicates (three containers per treatment) at regular intervals (0, 7, 14, and 21 days).

### 2.4. Biochemical Analysis

The biochemical analysis, including the quantification of total phenolic compounds, total flavonoids compounds, ascorbic acid (reduced and total content), and ORAC (oxygen radical absorbance capacity), were carried out on triplicates of broccoli samples following a series of methods described by Duarte-Sierra, Forney, Michaud, Angers, and Arul [23]. Briefly, total phenolic content determination was carried out by using the Folin–Ciocalteu method, assessed spectroscopically in a 96-well micro plate at an absorbance of 765 nm [25]. The rest of the assays were also evaluated spectroscopically in 96-well microplates. The total flavonoid content of florets was determined at 415 nm [26]; reduced ascorbic acid content was calculated at 525 nm [27]. ORAC measurements were performed at 37 °C with an excitation wavelength of 485 nm and emission wavelength of 530 nm [28]. All contents were expressed as g equivalent (gallic acid, quercetin, ascorbic acid, or Trolox) per kg.

### 2.5. Glucosinolates and Hydroxycinnamic Acid Analysis

The protocols by Duarte-Sierra, Forney, Michaud, Angers, and Arul [23,29] described the extraction, separation, and quantification of the glucosinolates (GLS) and hydroxycinnamates (HCA) of broccoli florets on a weight basis. The extraction consisted of combining 0.5 g of lyophilized samples from florets with 10 mL of 700 mL L^−1^ methanol and 800 µg on sinigrin (internal standard) at 70 °C for 10 min. The extracts were concentrated to dryness by evaporation and dissolved in 10 mM ammonium acetate/formic acid at pH 4.4. Compound identification was achieved using electrospray ionization MS on negative ion mode on a LC-MS (HP series 1100 LC/MSD) equipped with a 250 mm × 2 mm, 80 Å column (Phenomenex Synergi Hydro-RP) working at 30 °C. The separation of GLS and HCA was achieved by using retention times, and the identification was achieved by electrospray ionization MS on negative ion mode.

### 2.6. Gene Expression Analysis

The methodology for RNA extraction, cDNA synthesis, as well as the primers and their corresponding accession numbers, are described by Duarte-Sierra, Forney, Michaud, Angers, and Arul [23]. Reverse transcriptase polymerase chain reaction (RT-PCR) was carried out to analyze the expression of chalcone synthase (CHS), phenylalanine N-hydroxylase (CYP79A2), tryptophan N-hydroxylase (CYP79B3), dihomo-methionine N-hydroxylase (CYP79F1), flavonoid monooxygenase (F3H1), and phenylalanine ammonia-lyase (PAL) on day 0 after 6 h of storage at 4 °C.

### 2.7. Statistical Analysis

The statistical analysis was executed using the statistical analysis system version 9.3 (SAS Institute Inc. 2011. Base SAS^®^ 9.3 Procedures Guide. Cary, NC, USA). The analysis of data was carried out on a complete randomized design by one-way analysis of variance (one-way ANOVA) using a significance level of 0.05. The least significant difference test at the same significance level was performed when the analysis of variance found significant differences. The time-average value for total phenols, flavonoids, total ascorbic acid, and ORAC assay was calculated from day 0, 7, 14, and 21, and the result was used to compare ozone-exposed florets with non-treated broccoli.

## 3. Results and Discussions

### 3.1. Hormetic Dose of O_3_

**Figure 1:**
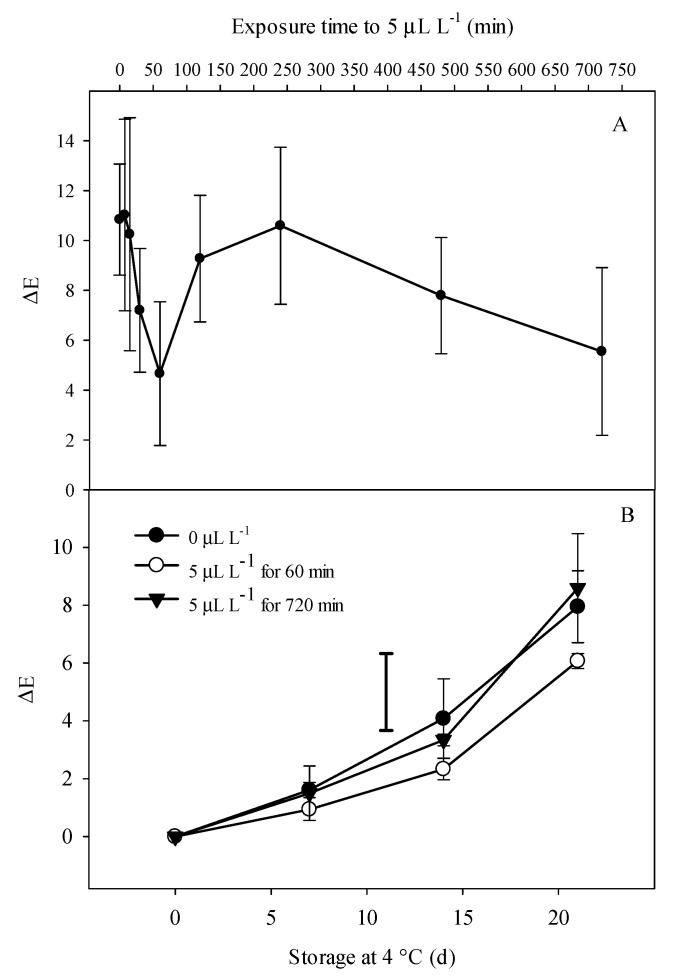
Ozone hormetic dose determination and color change of broccoli florets exposed to three different O_3_ doses. (**A**) Hormetic dose determination with 10 different O_3_ doses based on total color change of florets stored at 27 day at 4 °C/90–95%. (**B**) Total color change of (●) untreated florets; treated with (○) 5 µL L^−1^ for 60 min; and treated (▼) 5 µL L^−1^ for 720 min during 21 d at 4 °C/RH of 90–95%. Bars are the means of nine repetitions with +SD. The bar in graph B represents the LSD value (0.05) = 2.67. d, day.

The total color difference (ΔE) was monitored during storage to determine the hormetic dose of the ozone gas treatment. Among the exposure times to ozone at 5 µL L^−1^, the exposure of broccoli florets for 60 min was optimal for color retention (Figure 1A). Interestingly, the effect of ozone on the color retention of broccoli was bimodal (Figure 1A). The total color change (ΔE) was minimum after 60 min, with a ΔE value of 4.6, and a second minimum was registered after 720 min of exposure, with a ΔE value of 5.5 (Figure 1A).

The fumigation of produce with O_3_ is normally longer compared with ozonized water treatment [18], and hence, it is often carried out during the storage in the storage space, mainly to inhibit bacteria and fungi [14]. Nonetheless, O_3_ can also affect the physiology and the quality parameters of fruits and vegetables. For instance, floret opening and visual yellowing of broccoli exposed to continuous 0.04 µL L^−1^ of O_3_ for 21 d at 4 °C were significantly lower compared with the control, stored under the same conditions [30]. The color retention in celery was attributed to the inhibitory effect of ozonized water (0.03–0.18 ppm) on polyphenol oxidase activity (PPO) [31]. However, high doses of O_3_ can also cause phytotoxicity that is often characterized by discoloration and browning of the tissue [14]. Nonetheless, this may vary according to the method of application, for example, relatively high concentrations of O_3_ (up to 10 mg L^−1^ for 60 min) dissolved in water did not affect the quality of carrots treated with this element [32].

The fact that a bimodal behavior in the dose–color-retention relationship was present may be related with some mechanism operating to protect chlorophyll at high doses, or its accumulation functioning as an antioxidant. It is also possible that the degradation of carotenoids (e.g., lutein) renders chlorophyll more visible. Another possibility is that chlorophyll was converted into pheophytin due to the high weight loss in florets exposed to 5 μL L^−1^ of ozone for 720 min. In fact, this was evident at the end of the treatment, since the florets presented an olive-green coloration. However, this observation deserves further research, as chlorophyll A is more sensitive to ozone than chlorophyll B [5]. Yet, the conversion of chlorophyll A to pheophytin A displays a gray-brown color, whereas the transition of chlorophyll B to pheophytin B reveals olive-green colors [33].

### 3.2. Physiological Characteristics

#### 3.2.1. Color Evolution

**Figure 2:**
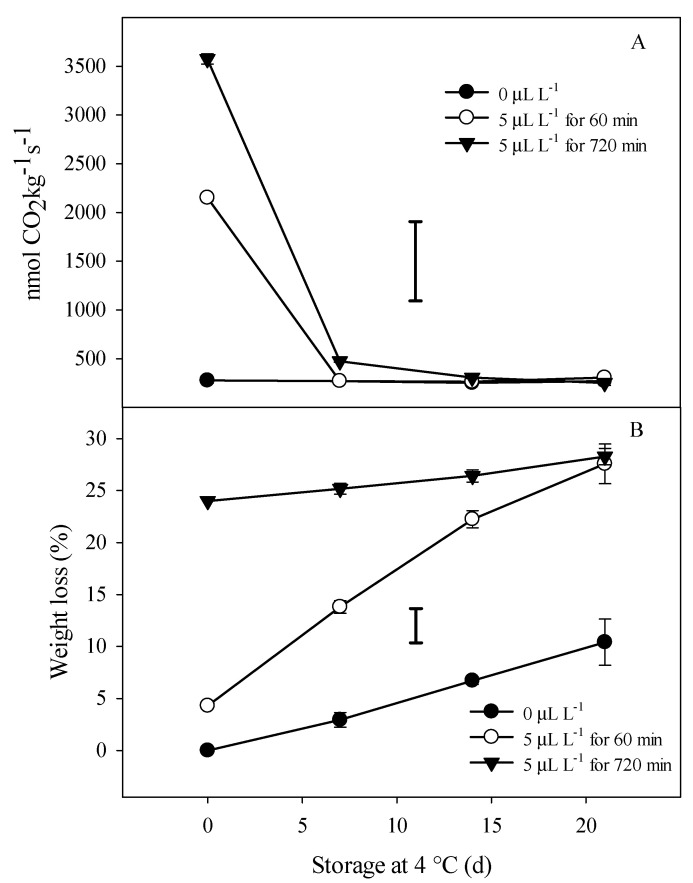
Progress of respiration rate and weight loss of O_3_-treated broccoli florets during storage of 21 d in the dark at 4 °C/90–95% RH. A: Production of carbon dioxide (CO_2_) of florets treated with three ozone doses: (●), control (0 µL L^−1^); (○), hormetic dose (5 µL L^−1^ for 60 min); and (▼), high (5 µL L^−1^ for 60 min) dose. Each point is the mean of three repetitions, and vertical bars represent standard deviation. B: Weight loss of florets during storage. Bars are the means of nine repetitions with ±SD. The bar in each graph represents the LSD value (0.05) = 813.3 (**A**) and 3.285 (**B**). d, day.

The color retention of florets exposed to the high dose of ozone was comparable to that of unexposed florets, but a significantly (*p* < 0.05) better color retention was observed in florets exposed to the hormetic dose of O_3_ at the end of the storage (Figure 1B). This was probably due, in part, to altered light reflectance characteristics, either because of the O_3_ reaction with cuticular waxes or because of the severe weight loss of O_3_-treated florets (Figure 2B). Long exposure to O_3_ may not only affect chlorophyll, but also carotenoids, especially lutein, which is responsible for the yellowing of florets, and thus, may reduce the yellowing of florets.

It is well known that the xanthophyll cycle, involving the epoxidation of zeaxanthin and the de-epoxidation of violaxanthin with the reductants, NADPH and ascorbic acid, respectively, protects the photosynthetic apparatus (thylakoids and chlorophyll) from oxidative stresses caused by drought, chilling, heat, senescence, and other abiotic stresses [34]. This cycle appears to be promoted in response to O_3_ exposure in tobacco leaves, where violaxanthin pool was reduced in tobacco, and that of zeaxanthin was slightly increased [35]. Immediately after exposure, violaxanthin de-epoxidation of zeaxanthin should be high, a substrate for ABA [35]. It would seem from the observation, where color change was more intense in the florets that were exposed to ozone for 240 min than in those exposed for 720 min, that the xanthophyll cycle was more operational in the latter, and that there was a build-up of reductive equivalents. These reductive equivalents can be transported to chloroplasts, where they can be used to reduce plastoquinone to plastoquinols [36].

#### 3.2.2. Respiration and Weight Loss

The respiration rate of the florets after the exposure to O_3_ was sharply higher compared to non-exposed florets (Figure 2A). The carbon dioxide production of the florets was 3744 nmol kg^−1^ s^−1^ following their exposure to 5 µL L^−1^ of O_3_ for 720 min, 2121.6 nmol kg^−1^ s^−1^ by the florets exposed to 5 µL L^−1^ of O_3_ for 60 min, and 174.72 nmol kg^−1^ s^−1^ by the untreated florets. By day seven, the high dose of ozone group exhibited a relatively high respiration rate of 499.2 nmol kg^−1^ s^−1^ compared with the rate of 224. 6 nmol kg^−1^ s^−1^ by the hormetic and untreated groups. After 14 days of storage, similar values were observed for the three treatments groups (250–300 nmol kg^−1^ s^−1^), without any significant difference (*p* > 0.05) between them.

Ozone gasification was a treatment in which the broccoli florets exhibited severe weight loss during exposure and during storage (Figure 2A). The weight loss of the florets during exposure to ozone for 720 min (high dose) was about 24% compared with 4% with the hormetic dose (exposure time of 60 min). However, the weight loss of high-dose ozone-treated florets during storage was small (24% to 28%), but the water loss of florets treated with the hormetic dose continued to increase during storage at a higher rate until it reached the weight loss of the high-dose group after 21 d of storage (27.5%). The weight loss during treatment cannot be solely attributed to moisture loss. Some other event, specific to ozone, occurred, which caused weight loss in addition to moisture loss, if any. The weight loss during storage is likely due, for the most part, to moisture loss, where the small or large increases in moisture loss can be attributed to the induction of osmolytes, such as proline, and surface morphological changes caused by ozone. Ozone is known to modify cuticular lipids, and as a result, the produce may develop thinner cuticles, leading to elevated moisture loss [14]. Surprisingly, some reports have noted that weight loss has been decreased in chili peppers [37] and pomegranates [38]. These observations may be due to the nature of the products, the pomegranate having a thick peel, and possibly, a very resistant variety of chili peppers.

The event during the exposure to ozone that causes severe weight loss, other than moisture loss, might be the emission of volatiles. The observation that the weight loss of the florets during exposure to ozone (in a high-humidity chamber) was very significant supports this possibility. The generation of volatiles in plants, ethylene, and isoprenes is well recognized [39,40,41], and may function as a detoxification mechanism.

### 3.3. Antioxidant Capacity of Florets

Antioxidant capacity was indirectly measured in broccoli florets by their content on ORAC, ascorbic acid, and by the total amount of phenols and flavonoids. The ORAC values of florets were generally reduced by both treatments of O_3_ (Table 1). The ORAC values of florets were reduced by 18% with the hormetic dose of ozone, and by 24% when the florets were exposed to 5 µL L^−1^ of O_3_ for 720 min, compared with the control florets. Though the content of total ascorbic acid on non-exposed florets and those exposed to the hormetic dose remained very similar, the titers of total ascorbic acid content in the treated broccoli were considerably reduced by 16% after exposure of the florets to 5 µL L^−1^ of O_3_ for 720 min. On the other hand, no significant differences were observed on the total content of phenolic compounds on the non-exposed and exposed florets to ozone. However, a significant increase of 32% was registered on the total flavonoid content of the florets exposed to the hormetic dose of ozone, and 20% on plant material exposed to the high dose, both compared to non-exposed broccoli florets.

The reduction of ORAC values suggests that the tissue was under an overall oxidative stress. It is increasingly recognized that the generation of ROS is a clear indication of systemic signaling and the elicitation of defenses in plants [20,24,42]. Ozone is soluble in water, leading to the formation of multiple ROS, O_2_^−^, H_2_O_2_, peroxyl radical, and other active O_2_ species [3,4,5,6,43]. To overcome this oxidative damage, plants and horticultural products use ascorbic acid to maintain the cell redox state, since it is the most abundant antioxidant in plant cells [44]. In addition, ascorbic acid has been related with phytohormone signaling networks, but to be active, it must be fully reduced [45]. The former remark might be related with the low content of reduced ascorbic acid measured in the florets exposed to the high dose of ozone (Table 1), and the deficiency of the enhancement of glucosinolates and hydroxycinnamic acids by this dose on the florets. Although reduced ascorbic acid is the main mechanism to overcome ROS generated by ozone, small molecules, such as phenols (flavonoids, among other compounds), can protect plant cells. This was the case of the flavonoid content in this experiment, which increased in the florets exposed to both doses. It is possible that the generation of less toxic phenoxy radicals might be radical-specific, but this requires further investigation.

### 3.4. Glucosinolates and Hydroxy-Cinnamic Acids

**Figure 3:**
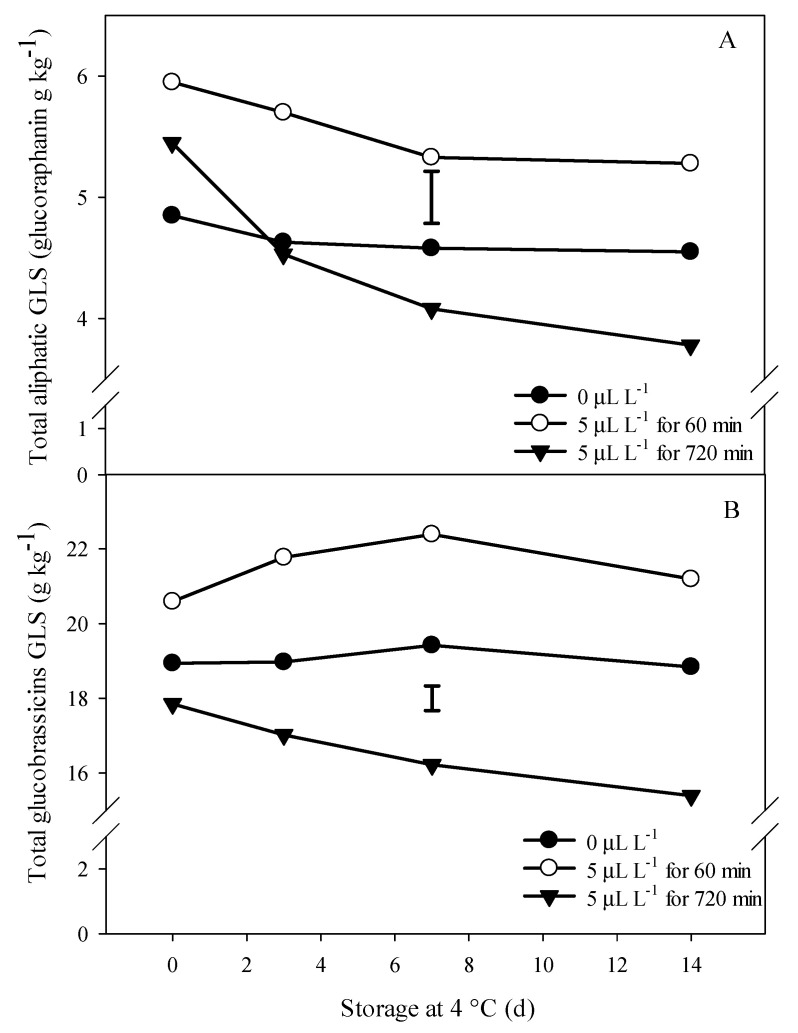
Effect of ozone exposure on total aliphatic and indole-type glucosinolates of broccoli florets. Total aliphatic (glucoraphanin) (**A**) and indole-type (**B**) glucosinolates in broccoli florets exposed to three doses of ozone, 0 µL L^−1^ (●), 5 µL L^−1^ for 60 min (○), and 5 µL L^−1^ for 720 min (▼), during storage in the dark for 14 days at 4 °C. Values are g equivalents of sinigrin on a dry weight basis. The bar in each graph represents the LSD value (0.05) = 0.54 (**A**) and 0.76 (**B**). d, day.

The enhancement of total aliphatic GLS and total glucobrassicins by O_3_ was observed only in the florets exposed to the hormetic dose, but with a significant decrease at the higher dose (Figure 3). The exposure of the florets to 5 µL L^−1^ of O_3_ for 60 min also increased the titers of total glucobrassicins by 13%; yet, the exposure of florets to the same concentration for 720 min decreased the titers by 12% (Figure 3).

**Figure 4:**
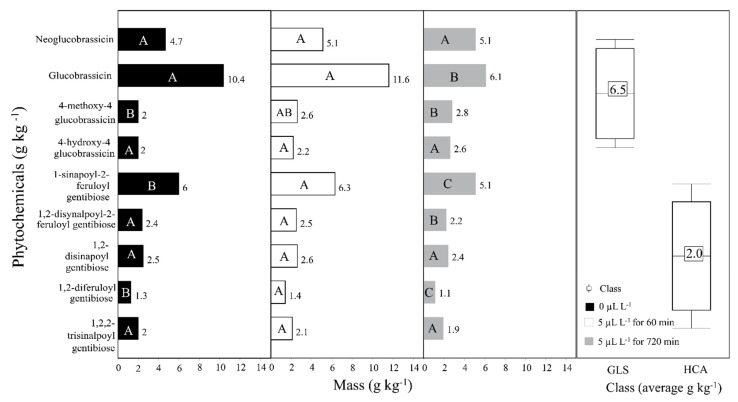
Ozone effect on individual glucosinolates and hydroxy-cinnamic acids. Florets were treated with O_3_ with 0 µL L^−1^, 5 µL L^−1^ for 60 min (hormetic dose), and 5 µL L^−1^ for 720 min (high dose), and stored for 14 days at 4 °C. Values are g equivalents of sinigrin on a dry weight basis ± SD (*n* = 3), and are the averages of 0, 7, 14, and 21 days. g kg^−1^. Different letters in each of the rows show significant differences (*p* < 0.05) between treatments according to Student’s *t*-test for each pair.

The same trend was perceived with the individual titers of GLS, where the overall amount of these compounds were more elevated compared to HCA content (Figure 4). The glucobrassicin concentration was found to be 11% superior with the hormetic dose of ozone in comparison to non-treated florets; however, it was drastically reduced by 41% as compared with the control (Figure 4). Further conversion of glucobrassicin to 4-methoxy-glucobrassicin in broccoli treated with ozone appeared to occur with the high dose, since its titer was 40% more elevated compared with the un-exposed florets (Figure 4).

**Figure 5:**
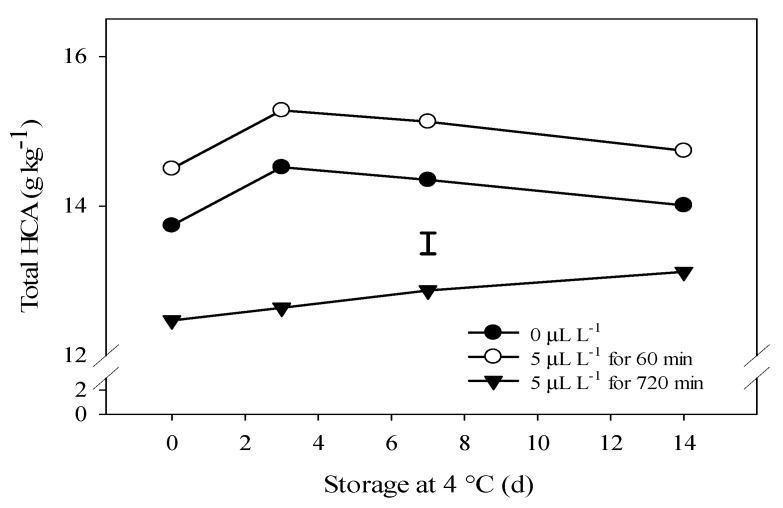
Broccoli florets were treated with three different doses of O_3_: (●), 0 µL L^−1^; (○), 5 µL L^−1^ for 60 min (hormetic dose); and (▼), 5 µL L^−1^ for 720 min (high dose). The content of total HCA was followed during 14 days. The vertical bar represents the LSD value (0.05) = 0.54. d, day.

Individual HCAs were also affected by ozone treatments following a general trend. Although not significant in most cases, all HCA in the florets were increased by the hormetic dose of ozone, whereas the high dose reduced the concentration of all of them (Figure 4). The most affected HCA was 1-sinapoyl-2-feruloyl gentibiose, where an increase of 5% was observed with the hormetic dose of ozone, but a reduction of 15% in the concentration of this compound was detected in the florets exposed to the high dose (Figure 4). This tendency was reflected on the total HCA content of the florets exposed to O_3_ (Figure 5). The total HCA content of broccoli was also reduced by 9% in the florets exposed to the high dose of O_3_. However, a small increase in the total HCA of 5% was observed when the florets were exposed to the hormetic dose of O_3_ (Figure 5). Interestingly, titers of HCA on the broccoli florets exposed to the high dose of ozone constantly increased during the storage period.

**Figure 6:**
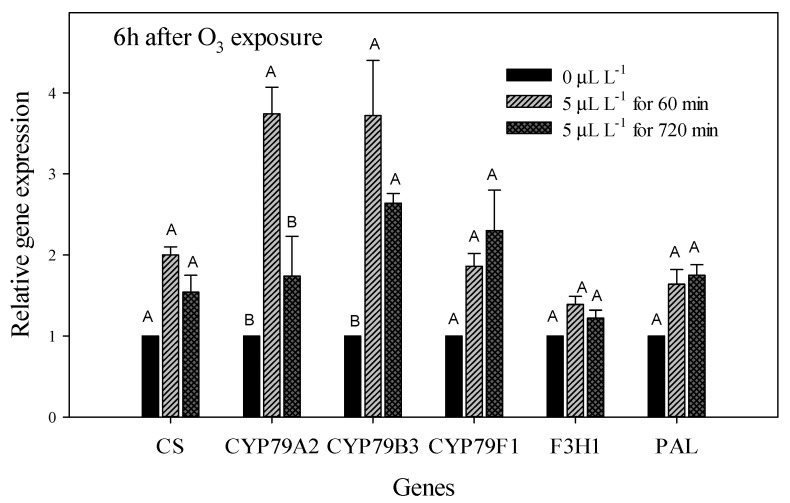
Gene expression analysis of broccoli florets exposed to O_3_ on day 0. Florets were treated with O_3_ at 0 µL L^−1^, 5 µL L^−1^ for 60 min (hormetic dose), and 5 µL L^−1^ for 720 min (high dose), stored at 4 °C/90–95% RH, and the values were normalized against actin. Gene expression in broccoli florets exposed to UV-B was measured after each treatment on chalcone synthase (CHS), phenylalanine N-hydroxylase (CYP79A2), tryptophan N-hydroxylase 2 (CYP79B3), dihomomethionine N-hydroxylase (CYP79F1), flavanone 3-hydroxylase (F3H1), and phenylalanine ammonia-lyase (PAL). Standard deviation is presented with vertical bars (*n* = 3). Different letters in each set of vertical bars show significant differences (*p* < 0.05) between treatments according to Student’s *t*-test for each pair.

In addition to the secondary metabolites analysis, the gene expression of key enzymes of the GLS and phenylpropanoid pathways were evaluated. The most affected gene expression corresponded to the GLS pathway. The high dose of O_3_ at 5 µL L^−1^ of O_3_ for 720 min amplified the expression of CYP79B3 by almost 4-fold, and the expression of CYP79F1 was increased by 2.5-fold (Figure 6). Furthermore, the hormetic dose of 5 µL L^−1^ of O_3_ applied for 60 min significantly (*p* < 0.05) increased the expression of phenylalanine N-hydroxylase (CYP79A2) by 4-fold compared to the control samples. Less significant results were observed with the phenolic pathway, where only the hormetic dose of ozone doubled the expression of chalcone synthase compared to the non-exposed florets (Figure 6). The levels of overexpression of GLS were in hand with the overall enhancement of total glucobrassicins and aliphatic GLS due to the hormetic dose of ozone (Figure 3). The same applied to HCA, where overall enhancements corresponded to the florets treated with the hormetic dose (Figure 5). Moreover, the overexpression of gene expression was also related to the average induction of each compound class, being superior on GLS (Figure 4).

The contribution of phenols, and specifically flavonoids, such as quercetin, to the detoxification of superoxide radicals produced by ozone in plants is limited [5]. Nonetheless, their accumulation seems to be a general response to low levels of this stress. In tomato fruit treated with a dose of 10 µL L^−1^ of ozone for 10 min, there was an increase in the total phenolic content by 50% compared to non-exposed fruit after 6 d of storage at 20 °C [46]. In fresh-cut papaya exposed to 9 µL L^−1^ of ozone for 30 min, there was an increase in the total content of phenols by 10.3% compared to the control fruit [15]. This trend was common in other commodities, such as kiwifruit exposed to 0.3 µL L^−1^ of O_3_ continuously for 4 months [47], and red peppers exposed to 0.1 µmol mol^−1^ [48]. However, it is not clear why the level of accumulation and the gene expression observed on the phenylpropanoid branch was lower compared to the build-up on the GLS pathway, especially the indole-type branch.

It has been suggested that the biosynthesis of more reduced compounds, such as GLS, compared to HCA, from their average carbon oxidation state (ACON), is favored when synthetic power (NADPH + H^+^) is available because of an oxidative stress event [24,49]. This seems to be the case for the hormetic dose of ozone. In hand with our results, higher doses of ozone have been reported to decrease the levels of GLS in *Brassica napus* exposed to 176 nL L^−1^ of O_3_/4 h/3 d [50], and *Brassica nigra* exposed to 120 ppb of O_3_ for 5 d [51]. Recently, Han, et al. [52], from a report on the effect of different O_3_ exposure durations on the plant growth and biochemical quality of *Brassica campestris* L. ssp. Chinensis, proposed a mechanism of GLS in response to O_3_. They suggest that the overall O_3_ response depends on the plant tolerance of ozone exposure accompanied by cell disruption. In Brassicas, this physical cell damage is normally followed by an accumulation of aromatic and indole-type individual GLS. Physical cell damage is also responsible for ROS rise and the concomitant increase of salicylic acid (SA) and jasmonic acid (JA). JA is precisely the plant signal involved in the synthesis of indole-type glucosinolates [53,54]; thus, the association between the gene expression of CYP79B3 and the titers of indole glucosinolates in ozone-treated broccoli florets suggests that the target of O_3_ is likely to be the pathway for indole glucosinolates.

## 4. Conclusions

The oxidative stress intensity, as manifested in the stress respiration, was significant with both doses of O_3_. Ozone also caused a significant weight loss during exposure of the florets even with the hormetic dose, and it was drastic at the high ozone dose. The hormetic dose of O_3_ was effective in elevating the levels of glucoraphanin and glucobrassicins, but not the titers of hydroxycinnamic acids. Furthermore, the high ozone dose depressed the levels of both glucosinolates and hydroxycinnamic acids; thus, from a commercial perspective, these doses need further optimization to satisfy quality requirements (weight loss). Ozone fumigation has been shown to induce phytochemical compounds related to human health at relatively low concentrations, and these concentrations remain constant for about two weeks, reflecting the actual marketing period of the vegetable. This study did not address the sensory aspects, but in the future, if this gas is to be used commercially, it will be necessary to determine whether the induction of these compounds negatively affects the taste and odor of the vegetable.

## Figures and Tables

**Table 1:** Oxygen radical absorbance capacity (ORAC, Trolox equivalents), and ascorbic acid (oxidized, reduced, total), total phenols (gallic acid equivalents), total flavonoids (quercetin equivalents), rutin (sinigrin equivalents), and chlorogenic acid (sinigrin equivalents) contents in broccoli florets exposed to ozone.

ORAC (g kg^−1^)			
0 µL L^−1^	172.28 ± 31.51 ^a^		
5 µL L^−1^ for 60 min	157.51 ± 20.21 ^ab^		
5 µL L^−1^ for 720 min	153.57 ± 20.86 ^b^		
Ascorbic acid (g kg^−1^)			
	Oxidized	Reduced	Total
0 µL L^−1^	3.40 ± 0.71 ^b^	8.20 ± 4.15 ^a^	11.66 ± 4.41 ^a^
5 µL L^−1^ for 60 min	3.25 ± 0.48 ^b^	8.98 ± 0.81 ^a^	11.92 ± 0.95 ^a^
5 µL L^−1^ for 720 min	4.08 ± 0.45 ^a^	5.27 ± 2.12 ^b^	8.51 ± 1.81 ^b^
Total phenols (g kg^−1^)			
0 µL L^−1^	15.33 ± 3.98 ^a^		
5 µL L^−1^ for 60 min	16.02 ± 3.60 ^a^		
5 µL L^−1^ for 720 min	14.31 ± 4.69 ^a^		
Total flavonoids (g kg^−1^)			
0 µL L^−1^	5.14 ± 1.74 ^b^		
5 µL L^−1^ for 60 min	7.23 ± 2.16 ^a^		
5 µL L^−1^ for 720 min	7.83 ± 1.59 ^a^		

Florets were treated with ozone at 0 µL L^−1^ (control), 5 µL L^−1^ for 60 min (hormetic dose), and 5 µL L^−1^ for 720 min (high dose), and were stored for 14 days at 4 °C/90–95% RH. The values + SD (*n* = 3) are the averages of 0, 7, 14, and 21 days. Each value represents the mean of three replicates along with standard deviation. Different letters in each of the columns show significant differences (*p* < 0.05) between treatments according to Student’s *t*-test for each pair.

## Data Availability

Data set can be found at: doi:10.17632/7rhxnv5xyb.1.
